# Laparoscopic inguinal hernia repair with self-fixated meshes: a randomized controlled trial

**DOI:** 10.1007/s00464-025-11616-5

**Published:** 2025-02-20

**Authors:** Anna-Maria Thölix, Jyrki Kössi, Marie Grönroos-Korhonen, Jukka Harju

**Affiliations:** 1 Department of Abdominal Surgery, University of Helsinki and Helsinki University Hospital, Helsinki, Finland; 2 Department of Surgery, Päijät-Häme Central Hospital, Lahti, Finland

**Keywords:** Inguinal hernia, Laparoscopic repair, Mesh fixation

## Abstract

**Background:**

Laparoscopic inguinal hernia surgery leads to rapid recovery and low complication rates. An alternative to fixate the mesh is using a self-fixated mesh.

**Methods:**

From April 2021 to June 2024, we conducted a randomized controlled trial comparing self-adhesive mesh (Adhesix™) with self-gripping mesh (Progrip™) in laparoscopic inguinal hernia surgery (TAPP and TEP). Adult patients scheduled for day surgery were included in the study with a 1-year follow up. The primary endpoint was the number of analgesics (Paracetamol or Ibuprofen) used during the first post-operative week. Secondary outcomes were pain-related issues, complications, and recurrence rate.

**Results:**

A total of 174 patients participated; 90 received Adhesix™ (group A) and 84 Progrip™ (Group P). Forty-six (26.4%) patients had recurrent hernia, 68 (39.1%) had unilateral and 60 (34.5%) had bilateral primary hernias. A total of 156 (90%) patients completed follow up. The number of analgesics during the first post-operative week was comparable between groups (P 22.9, A 21.2 tablets, *p* = 0.461). Group P used more analgesics during day 1, after which no difference was observed. In general, all participants used analgesics after surgery regularly for 10.8 days (SD 10.6) and occasionally for 15.9 days (SD 16.9). Time to return to work and normal activities was 16.1 days (SD 10.8) and 16.6 days (SD 9.6), respectively. More patients in group P reported moderate or severe pain (numeric rating scale > 3) during exercise 3 months after surgery (P 15.4%, A 3.1%, *p* = 0.035), although no difference was observed at 1 year after surgery. Both groups had significantly improved quality of life measures in physical aspects of the RAND-36 Item Health Survey after 3 months. Two recurrences, one in each group (1.1%) occurred.

**Conclusion:**

The use of Adhesix was non-inferior to Progrip in laparoscopic surgery. Surgery using either mesh led to rapid recovery and improved quality of life.

This trial was registered in ClinicalTrials.gov (NCT05091853).

**Graphical abstract:**

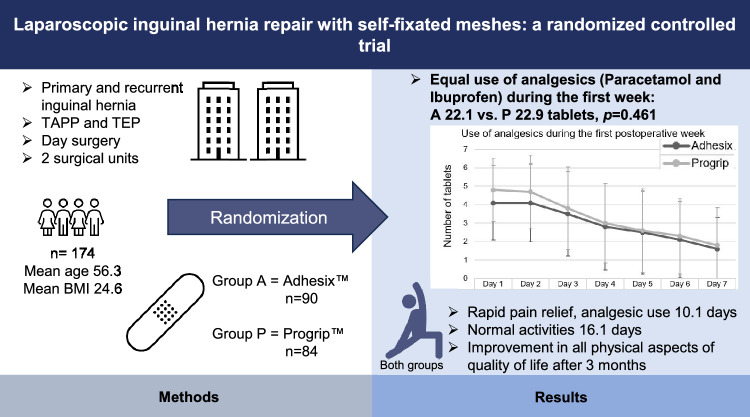

**Supplementary Information:**

The online version contains supplementary material available at 10.1007/s00464-025-11616-5.

Hernia in the inguinal area is fairly common; approximately 27% of males and 3% of females develop a groin hernia during their lifetime [1, 2]. Hernia treatment mainly involves surgery, with more than 20 million procedures performed annually [3, 4]. Multiple treatment options exist for inguinal hernia surgery, including open or laparoscopic techniques. According to guidelines, usage of mesh is recommended both in open and laparoscopic procedures as it reduces hernia recurrences. However, mesh is associated with both acute and chronic post-operative pain [4, 5].

Laparoscopic techniques include transabdominal preperitoneal repair (TAPP) and total extraperitoneal repair (TEP). In TAPP, the procedure is performed through the abdominal cavity, while in TEP the preperitoneal space is entered directly [6]. Both TAPP and TEP have good results with low complication and recurrence rates and rapid recovery after surgery [7–10]. Guidelines recommend both techniques for laparoscopic hernia surgery [4, 11]. Both techniques have comparable results regarding chronic pain and quality of life after surgery [7, 8, 12]. In laparoscopic procedures, mesh is placed in the preperitoneal space to cover the myopectineal orifice and posterior part of the inguinal area. The mesh can be fixated by tacks, glue or sutures. Alternatively, the mesh is not fixated at all or a self-fixating mesh can be used [13–15]. Studies recommend avoiding tacks, as they appear to cause more post-operative pain than glue or non-fixation [13, 16].

There are two different self-fixating meshes on the market, namely the self-gripping Progrip™ mesh and the glue-coated Adhesix™ mesh. The Progrip™ mesh has resorbable micro grips on a non-resorbable mesh, whereas Adhesix™ consists of a non-resorbable mesh covered by a self-adhering gel of polyvinylpyrrolidone (PVP) and polyethylene glycol (PEG) [17, 18]. The Progrip™ mesh has been used with better outcomes in laparoscopic inguinal hernia surgery compared with fixated meshes in several studies [13–15, 19]. There are only a few clinical studies using Adhesix™. Trials comparing these self-fixated meshes in laparoscopic surgery have not yet been published.

The use of self-fixated meshes in open hernia surgery demonstrated that patients experienced more acute pain after surgery with Progrip™ compared with Adhesix™ [20]. As there are no published randomized controlled studies comparing these two meshes in minimally invasive hernia surgery, it was thought that conducting such a study would be extremely important. The primary intention of the current randomized controlled trial was to evaluate post-operative pain after laparoscopic surgery, particularly in the early recovery period.

## Methods

### Trial design

This study was a prospective single-blind randomized clinical trial conducted in two surgical units in Finland, the Helsinki University Hospital and Päijät-Häme Central Hospital, from April 2021 through June 2024. There were two arms in the study. Patients were randomized at an allocation ratio of 1:1 to receive glue-coated self-adhesive mesh (Mesh Adhesix™, 10 × 15 cm, Cousin Biotech) or self-gripping mesh (ProGrip™ Laparoscopic Self-fixating Mesh, 10 × 15 cm, Flat Sheet, Covidien). This trial was approved by the ethics committee of Helsinki University Hospital (HUS/3413/2020) and registered in ClinicalTrials.gov (NCT05091853). This report adheres to the CONCORT 2010 guidelines [22].

### Participants

Enrolled participants were adult (≥ 18 years) patients with symptomatic inguinal hernia confirmed by clinical examination and suitable for operation in day surgery with laparoscopic technique (TAPP or TEP). Unilateral and bilateral operations were included for both primary and recurrent hernias.

Exclusion criteria included scrotal or incarcerated hernia, femoral hernia without an inguinal hernia finding, previous laparotomy, and no clinically palpable hernia. High-risk patients not suitable for day surgery, such as American Society of Anesthesiologists (ASA) physical status classification IV or more, body mass index > 35 kg/m^2^, liver cirrhosis, or other general illness causing contraindication for day surgery were not included. As the surgeon needed to be familiar with both meshes, patients were not enrolled in the study in case of teaching surgery where a surgical trainee performed the operation under supervision of a consultant. Other reasons for exclusion were inadequate language skills of the patient or if the patient declined to participate. Excluded patients were recorded.

Written informed consent was obtained from all participants.

### Procedures

The operations were performed under general anesthesia. The surgical technique (TAPP or TEP) was chosen by the surgeon. In case of TAPP, the abdominal cavity was entered with three trocars (one 12 mm and two 5 mm). A preperitoneal flap was created to achieve adequate exposure of the myopectineal orifice (MPO) and to facilitate correct mesh placement. Finally, the peritoneal flap was sutured. In the TEP, a small incision was made laterocaudally near the umbilicus and the preperitoneal space was initially created by balloon dilatation. After initial dilatation with round shaped balloon (OMSPDB1000, Medtronic, New Haven, CT), a 10-mm Hasson trocar was inserted into the preperitoneal space for telescope and further two 5-mm trocars were inserted as working ports. Dissection of preperitoneal space was continued with 5-mm graspers to properly expose the inguinal area for correct mesh placement as described above. The mesh was tightly rolled (Adhesix™) or folded (Progrip™) before brought in through the 12 mm trocar to the hernia site. The Adhesix™ mesh unfolds when letting go of the mesh and the thin fabric covering the mesh was removed, while the Progrip™ mesh was unfolded manually.

The surgeons performing the operations were general surgeons with 5 to 30 years of experience in laparoscopic hernia surgery. Helsinki University Hospital has a volume of 450 laparoscopic inguinal hernia surgeries annually. In Päijät-Häme Central Hospital annual volume of laparoscopic hernia operations is 100. Follow up was scheduled at 1 month, 3 months, and 12 months after surgery. At each timepoint, participants were sent a questionnaire specifically designed for the study. Questionnaires were collected by the investigating surgeons. All participant medical records were also reviewed. In case of noteworthy clinical problems, such as chronic pain, the participants were examined in the outpatient clinic.

### Primary and secondary outcomes

The primary outcome was the number of analgesics used during the first week after surgery. Paracetamol and Ibuprofen were routinely prescribed after surgery, opioid pain medication was used only if pain relief was insufficient otherwise. One tablet is equivalent to 600 mg ibuprofen or 1 g of paracetamol. Secondary outcomes were post-operative pain intensity, timing for ability to return to work after surgery, complications, and recurrence rate.

### Sample size

Sample size estimation was based on results of earlier trials and our internal evaluation [23]. According to our pilot evaluation, the mean consumption of analgesics during the first post-operative week was 16 tablets in a laparoscopic inguinal hernia operation (TEP) without mesh fixation. It was assumed that analgesic consumption after hernia operation with self-adhesive mesh is comparable with operation without mesh fixation. In our previous study consisting of openly operated patients, the consumption of analgesics was 26% more in the self-gripping mesh group. The hypothesis of the study was that use of self-gripping mesh causes more post-operative pain also after laparoscopic operations; it was estimated that using 6 tablets more during the first post-operative week would be a clinically significantly different finding. Assuming 80% power and an alpha level of 0.05, 148 participants would be required for the study. Considering an estimated dropout rate of 10%, it was estimated that 164 patients needed to be enrolled. An interim analysis was conducted halfway, with no significant differences between the groups.

### Randomization

Patients were enrolled consecutively and randomly allocated to receive either of the meshes used in the study. TAPP or TEP was chosen according to surgeon preference and separate randomization lists were created for both techniques and centers. Randomization was also stratified by hernia type (unilateral primary, bilateral, or recurrent hernia). A computer-based randomization list was generated with blocks of 10. Numbered and sealed opaque envelopes were opened by the operating surgeon just before the procedure. The participants were blinded to the choice of mesh.

### Statistical methods

Extracted data were analyzed using IBM SPSS statistics version 28. Numeric variables were tested for normality of distributions with the Kolmogorov–Smirnov test and are described as mean and standard deviation. Comparison of data between the groups was performed with independent samples *t* test or Mann–Whitney *U* test, and Wilcoxon signed-rank test for related samples. Categorical data are described as numbers and percentages. The χ^2^ test or Fisher’s exact test was used for comparisons of categorical data. A two-tailed value of *p* < 0.05 was considered as statistically significant.

## Results

**Fig. 1:**
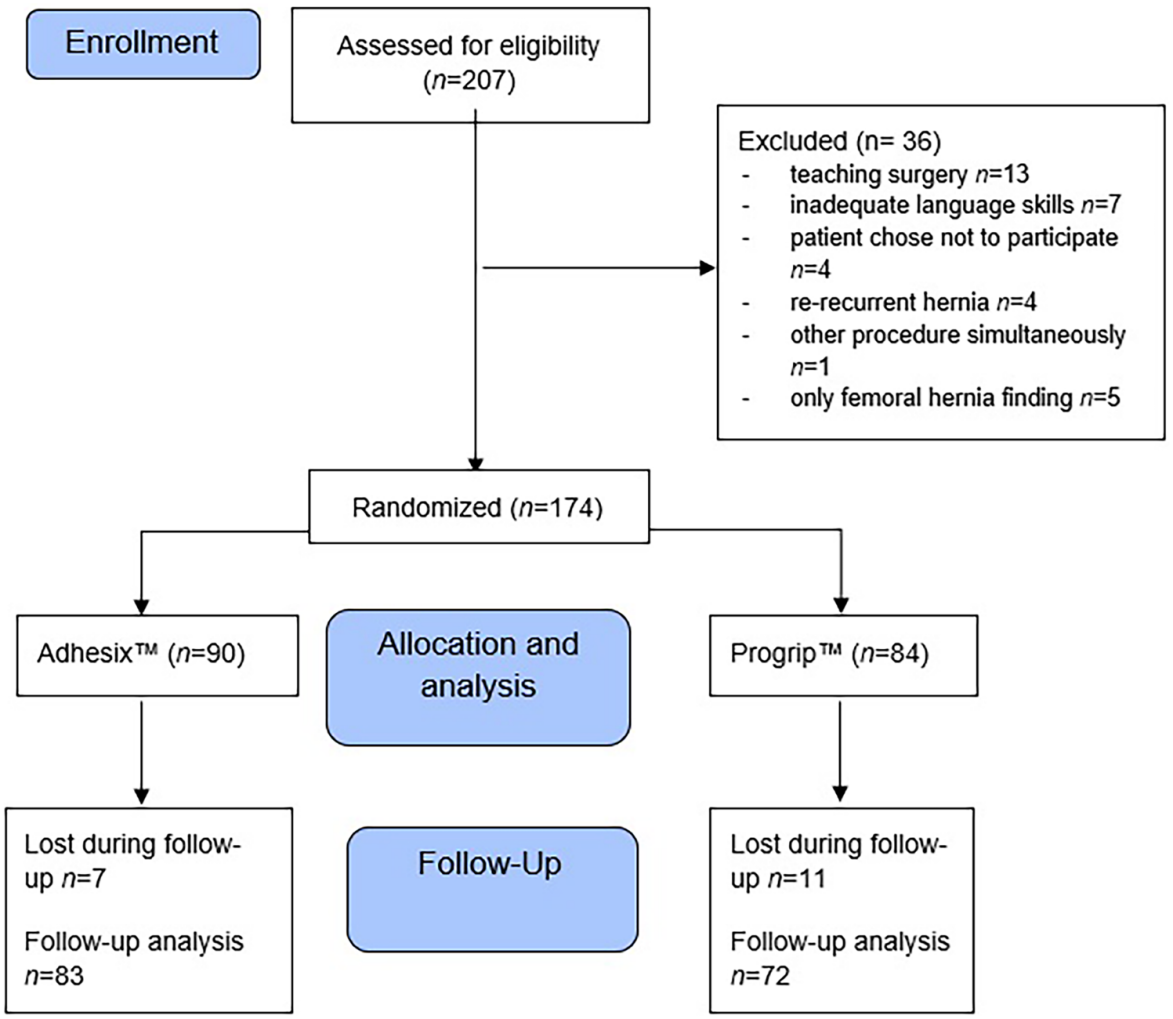
Flow diagram of the study design

Between April 2021 and June 2023, a total of 174 patients consented to participate in the study and were operated on. Ninety (51.7%) patients were randomly assigned to receive Adhesix™ mesh (group A); 84 (48.3%) received Progrip™ mesh (group P). A total of 156 (90%) participants completed follow up. Seven (7.8%) participants in group A and 11 (13%) in group P did not participate in the follow up. A flow diagram of the study is shown in Fig. 1.

### Patient characteristics

Participant demographics and characteristics are presented in Table 1. Mean age of the participants was 56 years (SD 14.1). Mean BMI was 24.6 kg/m^2^ (SD 3.24). Twelve patients (7%) had a BMI over 30. Proportionally more *n* = 5 (10.9%) of the patients with recurrent hernia were obese with a BMI over 30. Most (96%) participants were generally healthy (ASA score 1–2). These characteristics were comparable between groups. Group P included more females than group A (30 [35.7%] vs.18 [20%], *p* = 0.02). One quarter of the overall study population was female.

**Table 1:** Baseline characteristics

Baseline characteristics	All (*n* = 174)	Group A (*n* = 90)	Group P (*n* = 84)	*p*-value
Age (years), mean (SD)	56.3 (14.1)	58.4 (13.1)	54.1 (14.9)	0.043*
BMI (kg/m2), mean (SD)	24.6 (3.24)	24.5 (3.0)	24.7 (3.5)	0.758*
Male/female, n (%)	126 (72.4) / 48 (27.6)	72 (80.0) / 18 (20.0)	54 (64.3) / 30 (35.7)	**0.020**^**2**^
ASA classification, n (%)				0.558^2^
ASA1	83 (48.0)	42 (47.2)	41 (48.8)	
ASA2	83 (48.0)	42 (47.2)	41 (48.8)	
ASA3	7 (4.0)	5 (5.6)	2 (2.4)	

Bold p-value is statistically significant

p-Value: * = Student´s T-test, ^2^ = Chi-square.

### Operating details

Surgery in group P lasted longer. Mean operation time was 57 min (range 21–130 min) with Adhesix™ mesh and 65 min (range 25–142 min) with Progrip™ (*p* = 0.031).

A total of 135 (77.6%) TAPP and 39 (22.4%) TEP operations were performed; 128 (73.6%) participants were operated for primary hernias, of which 68 were unilateral and 60 bilateral. Forty-six (26.4%) participants had surgery due to recurrent hernias. There were no significant differences in the distribution of hernia type between the groups. One operation with the TEP technique was converted to TAPP and this participant was excluded. No other peri-operative complications were noted. Operative details are presented in Table 2.

**Table 2:** Operative details

Operative details	All (*n* = 174)	Group A (*n* = 90)	Group P (*n* = 84)	*p*-value
Op time, mean (SD)	61.3 (24.1)	57.4 (22.5)	65.3 (25.3)	0.031*
TAPP/TEP, n(%)	135 (77.6) /39 (22.4)	67 (74.4) / 23 (25.6)	68 (81.0) / 16 (19.0)	0.304^2^
Hernia type				
Primary/recurrent, n(%)	128 (73.6) /46 (26.4)	65 (72.2) / 25 (27.8)	63 (75.0) / 21 (25.0)	0.678^2^
Unilateral/bilateral, n(%)	114 (65.5) /60 (34.5)	57 (63.3) / 33 (36.7)	57 (67.9) / 27 (32.1)	0.530^2^
Hernia size (EHS)				0.915^2^
Direct (M1/M2/M3), n	86 (29/51/6)	51 (17/31/3)	35 (12/20/3)	
Indirect (L1/L2/L3), n	134 (51/67/16)	65 (17/39/9)	69 (34/28/7)	
Femoral (F1/F2/F3), n	3 (3/0/0)	1 (1/0/0)	2 (2/0/0)	
Combined	10	5	5	

Bold p-value is statistically significant

p-Value:* = Student´s T-test, ^2^ = Chi-square

### Primary outcome

**Fig. 2:**
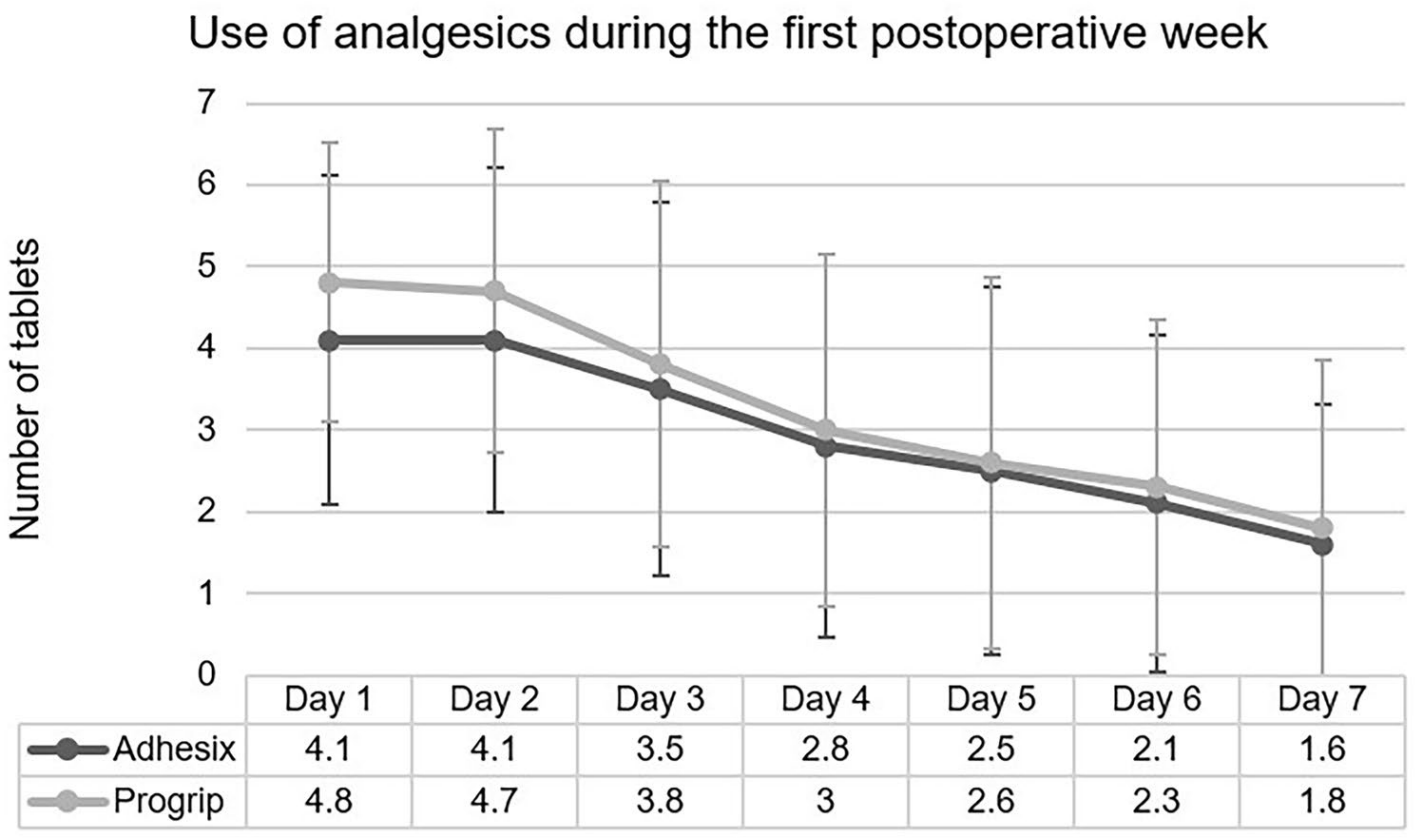
The number of analgesics needed during the first week after surgery

Primary outcome was use of analgesics during the first week after surgery. A total of 4.8% of the participants needed additional pain medication (tramadol or combination of paracetamol and codeine). Participants in group A used 21.2 tablets (SD 12.7) during the first week, whereas participants in group P used 22.9 (SD 12.8) tablets (*p* = 0.461). During the first post-operative day, group P used more analgesics than group A (4.8 vs 4.1, *p* = 0.027), after which no clear difference between groups was observed. Daily use of analgesics during the first week is presented in Fig. 2.

As the used operative technique could be an effect modifier, we performed a sensitivity analysis for the main outcome and found no statistically significant differences between techniques. Subgroup analysis for operative technique and hernia type are found in Supplementary File 2.

### Secondary outcomes

There were no statistically significant differences between groups in mean use of regular analgesics, mean occasional use of analgesics, and time to return to work or normal daily activities (Table 3*)*. Mean time for use of regular pain medication was 10.8 days (SD 10.6) after surgery. Mean time for use of analgesics, when necessary, was 15.9 days (SD 16.9). Before surgery, approximately one third of patients needed analgesics to relieve inguinal pain, whereas 17% (*n* = 20) and 12% (*n* = 16) used analgesics occasionally at 1 and 3 months after surgery, respectively. Participants returned to normal activities after mean 16.1 days (SD 10.8) and were fit for work after mean 16.6 days (SD 9.6). A total of 28% (*n* = 37) of the study population reported that they had not yet returned to all normal activities at 3 months after surgery.

**Table 3:** Pain-related outcomes

	All	Group A	Group P	*p*-value
Preoperative pain				
Preop use of analgesics				0.744^2^
Daily	9 (5.2)	4 (4.5)	5 (6.0)	
Irregularly	44 (25.4)	21 (23.6)	23 (27.4)	
No need	120 (69.4)	64 (71.9)	56 (66.7)	
Post-operative pain				
Number of analgesics (1st week), mean (SD)	22.0 (12.8)	21.2 (12.7)	22.9 (12.8)	0.461*
Regular use of analgesics (days), mean (SD)	10.8 (10.6)	12.0 (12.3)	9.3 (7.9)	0.171*
Irregular use of analgesics (days), mean (SD)	15.9 (16.9)	15.5 (16.7)	16.4 (17.3)	0.785*
Fit for work (days), mean (SD)	16.6 (9.6)	17.1 (10.1)	16.0 (9.0)	0.766°
Normal activities (days), mean (SD)	16.1 (10.8)	16.9 (13.0)	15.1 (7.2)	0.770°
Patients using opioid analgesics, n (%)	8 (4.6)	5 (5.6)	3 (3.6)	0.721^2^
No analgesic use at 1 month postop, n (%)	96 (82.8)	55 (87.3)	41 (77.4)	0.158^2^
No analgesic use at 3 months postop, n (%)	116 (87.9)	59 (88.1)	57 (87.7)	0.948^2^
Normal activities at 3 months postop, n (%)	95 (72.0)	47 (70.1)	48 (73.8)	0.636^2^
Unplanned vistits due to pain, n (%)	17 (9.8)	6 (6.7)	11 (13.1)	0.203^2^
Unplanned vistits due to complications, n (%)	18 (10.3)	8 (8.9)	10 (11.9)	0.621^2^

p-Value:* = Student´s T-test, ^2^ = Chi-square, ° = Mann Whitney U test

**Fig. 3:**
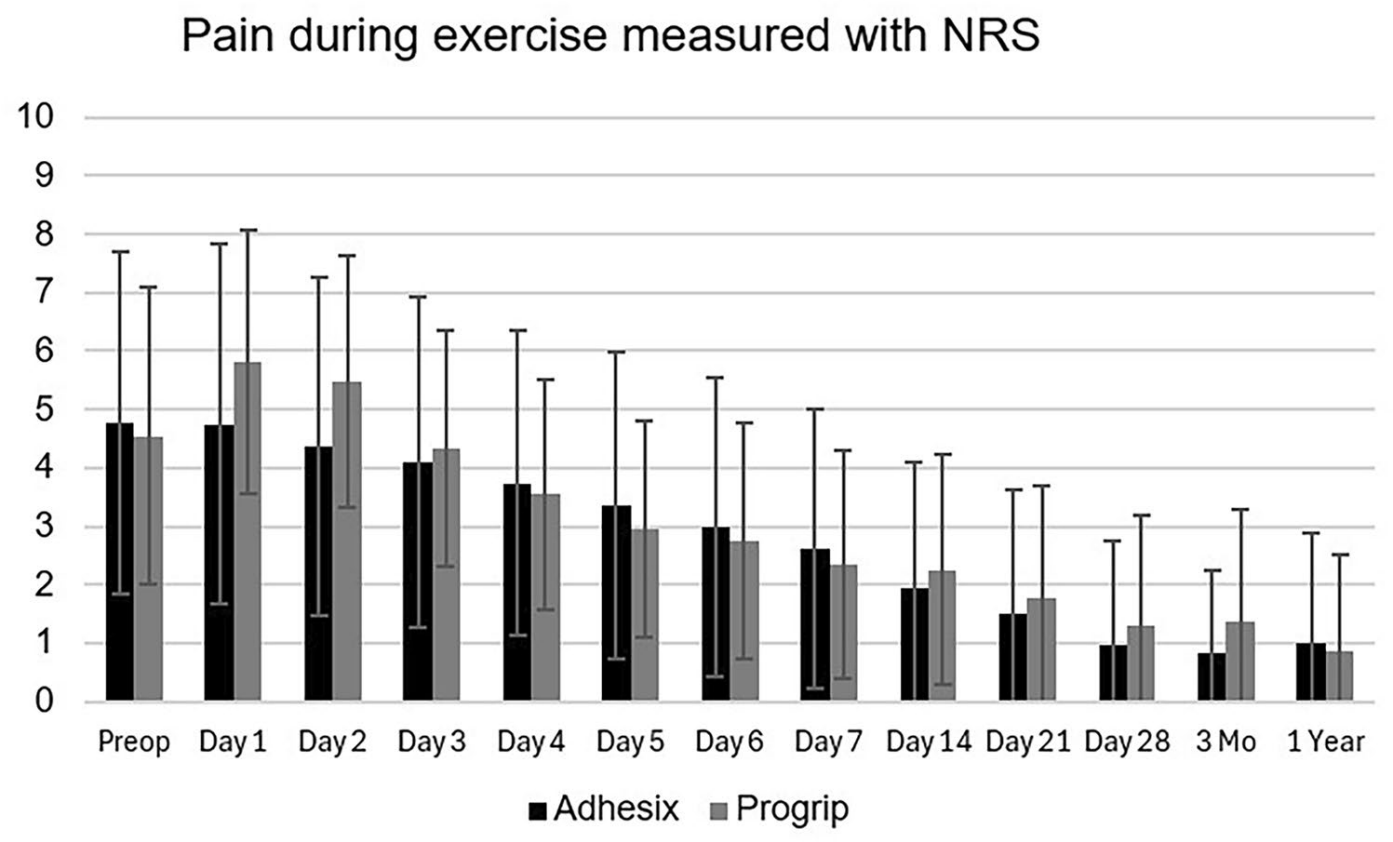
NRS (0–10) during exercise at pre- and postop follow-up

Pain intensity was measured using a numeric rating scale (NRS). Before surgery, mean NRS was 1.5 at rest, 2.5 when coughing, and 4.5 during exercise. At the first 2 days after surgery, NRS values reported by group P were higher than group A (Day 1: at rest 4.06 vs 2.91, *p* = 0.015; coughing 5.73 vs 4.79, *p* = 0.05; during exercise 5.82 vs 4.74, *p* = 0.048. Day 2: at rest 3.59 vs 2.52, *p* = 0.010; coughing 5.53 vs 4.32, *p* = 0.011; during exercise 5.48 vs 4.37, *p* = 0.021). After 2 days, both groups had comparable pain scores and had rapid pain relief. Figure 3 shows NRS values during exercise daily during the first week and at later timepoints until 1 year after surgery.

At 3 months and 1 year after surgery, the proportions of participants with pain at rest were unchanged, with around 85% of participants experiencing no pain at rest, 11% had mild pain, and approximately 3% intermediate pain. One participant (0.8%), which was in group P, reported severe pain at rest at 12 months after surgery. During exercise, group P reported more moderate or severe pain (*n* = 10, 15.4%) compared with group A (*n* = 2, 3.1%) (*p* = 0.035). In contrast, at 1 year 3 (5.6%) participants in group P and 8 (11.8%) participants in group A reported NRS value > 3 (*p* = 0.509).

**Fig. 4:**
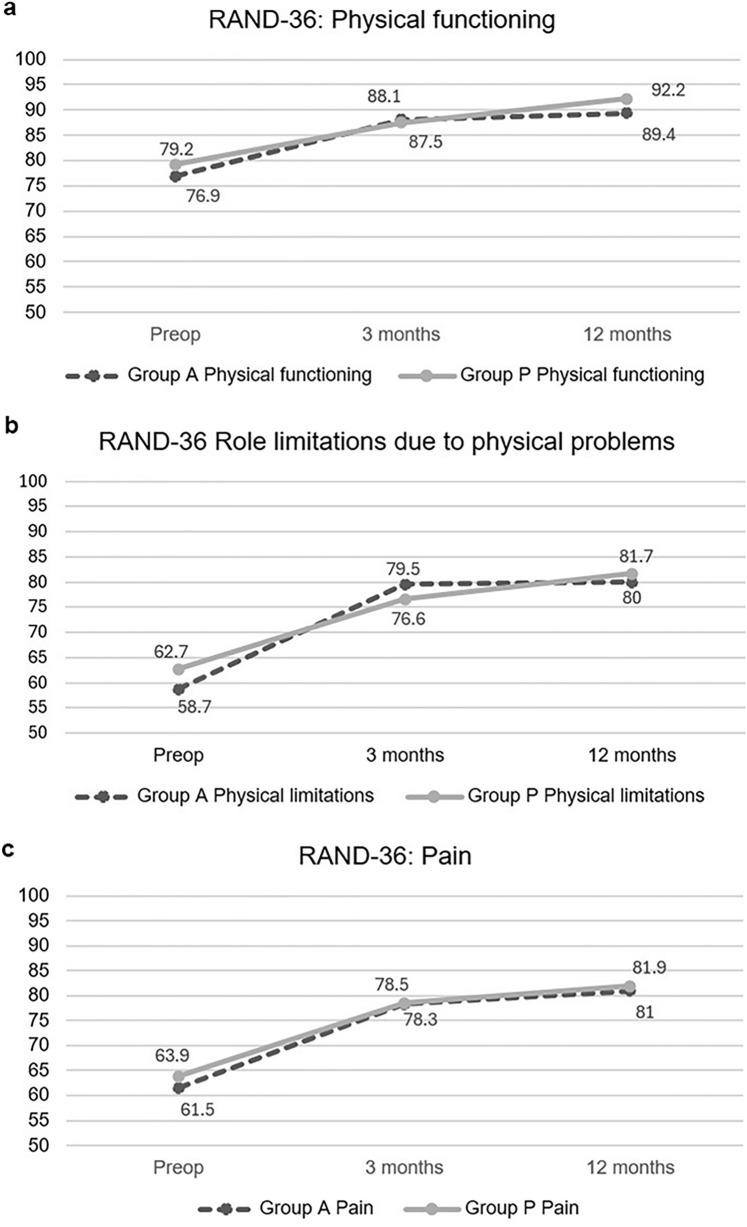
**a** Physical functioning measured with RAND-36 item health survey before surgery, three months and one year after surgery. **b** Role limitations due to physical problems measured with RAND-36 item health survey before surgery, three months and one year after surgery. **c** Pain measured with RAND-36 item health survey before surgery, three months and one year after surgery

To assess quality of life, the RAND-36 Item Health Survey [24] was completed by the participants before surgery and at 3 months and 1 year after surgery. Significant improvement was noted in all physical aspects (pain, physical functioning, and limitations due to physical problems) at 3 months after surgery compared with preoperative state. Additionally, social functioning was improved at 3 months after surgery. After 3 months, no additional significant improvement was observed in any aspect of the survey. Both groups had corresponding results (Fig. 4).

### Complications

Bruising in the inguinal area (*n* = 52, 43%) and the scrotal area (n = 41, 35.7%) were common. No participant needed reoperation or other intervention due to haematoma or bleeding. A total of 16% (*n* = 19) of participants reported seromas in the operated area, none of which needed treatment. No wound infections were reported needing antibiotic treatment. Only one reoperation was performed during the early recovery period due to seroma that was mistakenly thought to be a recurrent hernia. Two (one in each group) hernia recurrences occurred during the 1-year follow up, both later treated with open Lichtenstein mesh repair.

In case of normal recovery after surgery, participants had no clinical follow up at the outpatient clinic. Twenty-two (18.2%) participants needed a physician’s evaluation during the first month after surgery due to prolonged pain, sensory disturbances in the operated area or thigh, or swelling in the groin or scrotum. Some patients needed more effective analgesics (4.8% needed opioid analgesics, such as tramadol or combination of paracetamol and codeine) or prolonged absence from work, but no surgical interventions were required due to these problems. During the first year after surgery, one fifth (*n* = 34, 19.5%) of the operated participants needed an evaluation due to post-operative concerns (acute or chronic pain, 3 cases of hydrocele, 2 recurrences), with no significant difference between groups.

## Discussion

This is the first randomized controlled trial comparing the Progrip™ and the Adhesix™ self-fixating meshes in laparoscopic groin hernia surgery. In contrast to our previous findings in open inguinal hernia procedures, the current study did not reveal differences in many aspects of post-operative pain, such as analgesics used during the first week after surgery, the need for unplanned visits due to pain, and returning to normal activities and work. As expected after laparoscopic surgery, the average pain relief was rapid and quality of life was increased.

To our knowledge there are no previous trials comparing self-gripping and self-adhesive glue-coated meshes. However, in two randomized studies the use of self-gripping mesh has been compared with fibrin glue fixation. Law et al. reported similar results for both short- and long-term post-operative pain for the self-gripping Progrip mesh compared with glue fixation in TEP [15]. Ferrarese et al. reported similar results for TAPP in an otherwise identical setup [14]. There is only one published clinical trial on Adhesix™ in laparoscopic inguinal hernia surgery. In a prospective study by Tollens et al., the post-operative pain measured by visual analogue scale was mild at 1 month and long-term [25]. The findings of our study are consistent with this and it can be concluded that Adhesix™ is comparable with glue fixation of mesh. Furthermore, the benefits of Adhesix™ may be similar to the benefits of glue fixation published in the literature, such as less early post-operative pain [13–15]. As the overall results of the glue-coated mesh and glue fixation of mesh are comparable, a potential advantage of the glue-coated mesh may be that additional time-consuming and expensive fixation is not needed. A discrepancy between our previous and current study regarding post-operative pain is not evident. However, one explanation may be that in an open procedure, the contact of inguinal nerves to the mesh is more extensive and in the early recovery period, micro grips cause more irritation than glue.

There are two previous RCTs reporting post-operative NRS pain scores after TEP. Matikainen et al. reported NRS values of just below 2 at 1 week after surgery with TEP for unilateral primary hernias [26]. Corresponding results were reported by Yildrim and Sahiner [27]. The design of these studies was different than our study. In both studies, only patients with unilateral primary hernias were enrolled and they used polypropylene meshes (12 × 15 cm), which were not fixed at all. In the current study, low NRS scores, although slightly over 2, were noted at 1 week. The fact that approximately 60% of all hernias in our study were bilateral or recurrent reflects our routine practice, which according to the literature may lead to more post-operative pain [28]. This may explain our slightly higher NRS values. However, some benefits regarding NRS values for Adhesix™ were noted. At day 1 and 2 after surgery, group P reported higher pain scores, although during the subsequent days the difference was no longer observed. Interestingly, this did not reflect the amount of analgesics used during the first week, indicating that this small difference in NRS values is not clinically meaningful. The reason for this difference is not evident. However, one explanation could be that after tissue contact Adhesix™ mesh softens very rapidly causing less irritation compared to rigid micro grips containing and stiff Progrip™ mesh.

Although mean NRS after 3 months was low for both groups (0.8–1.3), some patients still experienced pain and discomfort for a longer time. A total of 28% in this trial reported some limitations in normal activities 3 months after surgery. When the numbers of participants experiencing moderate or severe pain during exercise at 3 months after surgery were considered, we found significantly more participants in group P than in group A (15.4% vs. 3.1%, *p* = 0.035). However, 5.6% in group P and 11.8% in group A reported NRS over 3 during exercise at 1 year, whereas at rest only 1.8–2.9% experienced intermediate or severe pain. As chronic pain is defined as at least moderate pain lasting more than 3 months after surgery and affecting daily activities [2, 29], we suggest that the proportion above gives an indication on chronic pain rates in this study. In the RCT using Adhesix™ and Progrip™ in open inguinal hernia surgery for unilateral primary hernias, the corresponding chronic pain rates (NRS > 3) at 1 year after surgery were 5.2–7.4%, with no statistical difference between the groups [21]. Other RCTs reported chronic pain rates of 4.5–11% one year after laparoscopic operations, which are consistent with our results [30–32].

The time to returning to normal activities and being fit for work were estimated by the participant in this trial, with no differences observed between groups. This is highly dependent on the participant’s own requirements and are not directly comparable between different trials. One RCT [26] with TEP revealed approximately 14 days for returning to work and normal activities, whereas other RCTs reported 10–14 days after laparoscopic surgery and 12–19 days after open mesh repair to return to work and normal activities [9, 15, 31]. In our study this took a few days longer, approximately 16 days, to reach working ability and normal daily activities. This discrepancy is probably explained at least in part by the different patient populations since, as mentioned earlier, most of our participants had bilateral or recurrent hernia. In contrast to the current study, in open hernia surgery using self-fixated mesh, the return to normal activities was significantly earlier in operations performed with Adhesix™ compared with Progrip™ (17 and 22 days, respectively) [20]. This finding may indicate that self-gripping Progrip™ mesh may be more suitable for laparoscopic hernia operations. Operation time was shorter in group A (57 min) compared to group P (65 min). This difference in time may partly be explained of the fact that the Adhesix mesh unfolds when letting go of the mesh while the micro grips in Progrip mesh grab to each other and needs to manually be unfolded.

Previous studies have shown rapid improvement in quality-of-life measures after laparoscopic surgery [8, 33]. In fact, patients experiencing more preoperative pain usually have more physical limitations before surgery and thus benefit the most in quality-of-life measurements [34]. The RAND-36 Item Health Survey [24] was used in this study. Significantly higher scores were reported at all physical areas already at 3 months after surgery, indicating rapid recovery after surgery. Similar conclusions were made after open surgery using the same meshes [21].

In this study, in addition to pain-related contacts, approximately a tenth of participants contacted a physician due to some kind of post-operative adverse event. This was mostly due to post-operative swelling or a lump in the area caused by hematoma, seroma, or recurrence. Except for two recurrences that required reoperation, no other interventions were needed. Although bruising in the inguinal area or scrotum is very common after inguinal hernia surgery, the need for interventions due to bleeding or hematoma was rare. Other studies report re-operations due to these complications (approximately 1% after open surgery), while laparoscopic surgery does not seem to need re-operations [26, 27, 31]. In our study, over one third of the participants suffered from bruising but no participants needed reoperation. Seroma formation is not rare after inguinal hernia surgery. The incidence of seroma is 7.2–38% after laparoscopic surgery, which is partly dependent on time of follow up [35, 36]. In the current study, 16% of patients reported fluid collections in the operated area during the first month. Two recurrences (1.3%) were observed in this study, which corresponds with other reports [27, 31, 32]. However, it should be noted that incidence of recurrence increases over time [37, 38]. Obesity is a risk factor for hernia recurrence [39] and also in this study higher BMI levels were noted in those operated for recurrent hernia. Particularly for the Adhesix™ mesh, no firm conclusions regarding recurrence rate can be made without longer follow up.

This study has some limitations. First, many factors influence the experience of pain, and an objective measurement of pain is difficult. Therefore, we can find differences in some measures while other measures are similar. Second, most participants were not examined after surgery. Although some minor problems may therefore have been undetected, participants with any clinically relevant problem were evaluated in the outpatient clinic. Additionally, we have a national electronic health care database allowing follow up 1 year after surgery. Third, some participants did not participate in the follow up, which may have influenced the results. The strength of this study was its randomized controlled design, with comparable follow up between groups and a high response rate of 90%. Our hypothesis that glue-coated mesh causes less acute pain measured by the amount of pain medication used, based on the superiority of Adhesix™ in our earlier trial on open hernia surgery, could not be confirmed in a laparoscopic setting.

For future research, long-term results after self-adhesive mesh have not yet been published and are therefore of great importance to be studied. As self-fixated mesh makes the operation more efficient it will be interesting to see if alternatives to glue or resorbable micro grips that can be developed and integrated in mesh fixation. Additionally, technical improvements, particularly robot assisted hernia surgery, show promising results, and its influence on post-operative pain is an important aspect to investigate in the future.

## Conclusion

This trial found that self-adhesive Adhesix™ mesh was non-inferior to self-gripping Progrip™ mesh in laparoscopic surgery. Surgery with either self-fixated meshes led to rapid recovery and quality of life improvement.

## Supplementary Information

Below is the link to the electronic supplementary material.

Supplementary file1 (DOCX 15 KB)

Supplementary file2 (DOCX 15 KB)

Supplementary file3 (DOCX 25 KB)
