# Supplementary material for: Laparoscopic inguinal hernia repair with self-fixated meshes: a randomized controlled trial
Source: Surg Endosc. 2025 Feb 20;39(4):2425–35. doi: 10.1007/s00464-025-11616-5 (PMC11933136; doi:10.1007/s00464-025-11616-5)
Supplement: Supplementary file 1 — Supplementary file1 (DOCX 15 KB) [file 464_2025_11616_MOESM1_ESM.docx]

| **Complications** | **Clavien-Dindo grade** | **Total (n=174)** | **Adhesix (n=90)** | **Progrip (n=84)** | **P-Value** |
| --- | --- | --- | --- | --- | --- |
| Inguinal bruising, n (%) | I | 52 (43.0) | 24 (35.8) | 28 (51.9) | 0.097² |
| Scrotal bruising, n (%) | I | 41 (35.7) | 25 (39.1) | 16 (31.4) | 0.437² |
| Seroma, n (%) | I | 19 (15.7) | 8 (11.9) | 11 (20.4) | 0.220² |
| Reoperation due to seroma (suspected recurrence), n | IIIb | 1 | 1 | 0 |  |
| Surgical site infection, n (%) | | 0 | 0 | 0 |  |
| Recurrence, n (%) | IIIb | 2 (1.1) | 1 (1.1) | 1 (1.2) | 1.000² |

Supplementary table 1. Complications. *p*-Value: ²= Chi-square
