# Supplementary material for: Laparoscopic inguinal hernia repair with self-fixated meshes: a randomized controlled trial
Source: Surg Endosc. 2025 Feb 20;39(4):2425–35. doi: 10.1007/s00464-025-11616-5 (PMC11933136; doi:10.1007/s00464-025-11616-5)
Supplement: Supplementary file 2 — Supplementary file2 (DOCX 15 KB) [file 464_2025_11616_MOESM2_ESM.docx]

| **Operative technique** | **TAPP n=135** | **TEP n=39** | ***p*-Value** |
| --- | --- | --- | --- |
| Group A |  |  |  |
| Number of tablets, n (SD) | 22.2 (12.7) | 18.4 (12.9) | 0.195* |
| Group P |  |  |  |
| Number of tablets, n (SD) | 24.8 (13.8) | 17.1 (8.0) | 0.060* |
| **Hernia type** | **Primary hernia n=128** | **Recurrent hernia n=46** | ***p*-Value** |
| Group A |  |  |  |
| Number of tablets, n (SD) | 21.5 (12.8) | 20.3 (12.9) | 0.718* |
| Group P |  |  |  |
| Number of tablets, n (SD) | 25 (12.5) | 16.5 (11.7) | 0.028* |

Supplementary file 2. Subgroup analysis for analgesic use during the first week after surgery. *p-Value:*=Student´s T-test*
