# Supplementary material for: Laparoscopic inguinal hernia repair with self-fixated meshes: a randomized controlled trial
Source: Surg Endosc. 2025 Feb 20;39(4):2425–35. doi: 10.1007/s00464-025-11616-5 (PMC11933136; doi:10.1007/s00464-025-11616-5)
Supplement: Supplementary file 3 — Supplementary file3 (DOCX 25 KB) [file 464_2025_11616_MOESM3_ESM.docx]

## Laparoscopic Inguinal Hernia Repair - Does The Choice Of Self-fixated Mesh Matter?

Version 1.1 January 25th, 2021

### Research group

Principal investigator: Anna-Maria Thölix, MD, Helsinki University Hospital, Abdominal center

Other members: Jukka Harju, MD PhD, Helsinki University Hospital, Abdominal center

Tom Scheinin, MD PhD, Helsinki University Hospital, Abdominal center

Jyrki Kössi, MD, PhD, Päijät-Häme central hospital

Research site: Helsinki University Hospital, Abdominal center, Day surgery and Päijät-Häme central hospital, Day surgery.

### Background

Inguinal hernia causing symptoms is treated with either open or laparoscopic surgery. According to international guidelines (HerniaSurge Group 2018) laparoscopic surgery is preferable in females and bilateral hernias. Additionally, in males with unilateral hernia laparoscopy is preferable if the surgeon has experience with the technique.

Transabdominal preperitoneal repair (TAPP) ja totally extraperitoneal repair (TEP) are today standard laparoscopic techniques (HerniaSurge Group 2018). Both techniques have equal results in complication rates and hernia recurrence (Köckerling 2015).

Acute and chronic pain after surgery are important criteria for successful treatment. Acute pain is the most important factor influencing convalescence after surgery. Chronic pain on the other hand is a challenging issue affecting quality of life. Many factors influence post-operative pain. Four to 12% suffer from chronic pain after inguinal hernia surgery with mesh, although laparoscopic surgery seems to cause lower pain rates compared to open surgery (Scheuermann, 2017).

Operative technique, the type of mesh used and mesh fixation may influence pain after inguinal hernia surgery. However, previous research on this topic is contradictory.

In the TAPP and TEP techniques the mesh is placed in the preperitoneal space. The mesh may be fixated with tacks or glue, left without fixation or self-fixated mesh can be used. Previous studies have mainly focused on different fixation methods and their effect on pain compared to non-fixation. Lin et al (2017) reported less acute and chronic pain and faster recovery after using mesh without fixation in small (<4cm) hernias. A randomized trial with 93 patients compared glue fixation to tacks in TEP operations on bilateral hernias and showed less pain after glue fixation (Lau 2005). Also, a meta-analysis by Sajid et al found less chronic pain after glue fixations compared to tacks without differences in recurrence rates (Sajid 2013). A more recent meta-analysis reported similar conclusions for post-operative morbidity (Shi 2017). Also, non-fixation shows similar results in operative time, time for recovery and post-operative pain. Non-fixation does not seem to cause more recurrence (Sajid 2012).

Only a few studies on self-fixated mesh in laparoscopic surgery have been published. A randomized trial with 100 participants did not report any difference in recovery or chronic pain rates when comparing self-fixated mesh with glue fixation (Cambal 2012). Another study compared self-fixated mesh to fixation with tacks in 96 patients and could not find any difference in recurrence and chronic pain rates (Romario 2013). Two prospective studies showed only minor complications and post-operative pain, and low recurrence rates in laparoscopic surgery with TEP (Ozmen 2015) and TAPP (Birk 2012).

Our own retrospective study (Thölix 2018) suggested less problematic post-operative pain after open inguinal hernia surgery with self-adhesive (Adhesix™) compared to self-gripping (ProGrip™) mesh. Laparoscopic inguinal hernia surgery comparing similar self-fixated mesh have yet not been published.

### Objective

Pain after inguinal hernia surgery impacts the patient´s well-being and quality of life. The goal for this trial is to examine if the choice of mesh affects pain after laparoscopic hernia surgery.

Two self-fixated mesh are compared in this study on surgery with laparoscopic techniques. The participants are randomized in two groups. One group get the self-adhesive glue coated Adhesix™ mesh whereas the other group get the self-gripping ProGrip™ mesh, with resorbable micro grips, in an otherwise routine laparoscopic operation. Both meshes are routinely used in todays practice.

Main outcome is the use of analgesics during the first week after surgery.

### Participants

*Inclusion criteria:*

Adult (>18 year) patients with a clinical inguinal hernia getting laparoscopic surgery treatment in the day surgery unit.

*Exclusion criteria:*

Scrotal or incarcerated hernia

Previous laparotomy

ASA classification >3

BMI <18 or >35

Liver cirrhosis

No hernia in clinical examination

The participants receive thorough oral and written information about the study before enrollment. The document “Information for the patient” is included. Participation in the study is voluntary, and the patient will have sufficient time to consider participation. Written informed consent is obtained from all participants.

### Methods

This is a randomized controlled trial. Data is collected from the medical records, data collection forms and questionnaires. Collected data are analyzed and published in international medical journals.

A routine laparoscopic inguinal hernia operation (TAPP or TEP) is carried out in the day surgery unit. The mesh used is randomized. A computer-based randomization list is generated in blocks of 10. Numbered and sealed envelopes containing the patient´s study group are opened by the operating surgeon just before the procedure. The procedure is done under general anesthesia. The patients receive normal post-operative instructions, Paracetamol and Ibuprofen are routinely prescribed for post-operative pain. Sick leave for work absence is one week or in case of physically demanded job two weeks.

Normal follow-up is applied, including clinical examination in case of post-operative problems. The participant fills in a pain related questionnaire reporting daily pain and analgesics use during the first week after surgery, after which weekly documentation until one month after surgery. At 3 months and one year after surgery the patients reports data about pain and recovery. Also, participants´ quality of life is assessed using the RAND-36 Item Health Survey before surgery, at 3 months and one year after surgery.

The main outcome for this trial is the number of analgesics used during the first post-operative week. Secondary outcomes are post-operative pain intensity, timing for ability to return to work after surgery, complications, chronic pain and recurrence rate.

### Timeline

The study is planned to begin 1.1.2021. Data collection is estimated to last around two years, after which patients are followed up for one year after surgery.

### Sample size and statistical methods

Sample size estimation is based on results of our internal evaluation and an earlier quite similar trial (Lau, Ann Surg 2005), assuming 80% power and an alpha level of 0.05. According to our pilot evaluation, the use of analgesics during the first week after surgery was 16 tablets in a laparoscopic inguinal hernia operation without mesh fixation. We assume that analgesic consumption after hernia operation with self-adhesive mesh is comparable with operation without mesh fixation. The hypothesis of the study was that use of self-gripping mesh causes more postoperative pain also after laparoscopic operations; it was estimated that using 6 tablets more during the first postoperative week would be a clinically significantly different finding. 148 participants are needed for the study. With an estimated dropout rate of 10%, we will enroll 164 patients.

Comparison of data on numeric variables will be performed with independent samples *t* test or Mann-Whitney *U* test, and Wilcoxon signed-rank test for related samples. Categorical data are analyzed with the χ^2^ test. Data is analyzed using IBM SPSS statistics.

An interim analysis will be conducted halfway to see if statistical differences are found between the groups in main outcome or adverse events.

### Handling of data

Data collected up to the time of termination will be analyzed.

### Adverse events

Patient care (diagnostics, operative care and follow-up) is performed as standard hospital practice. The meshes used in the trial are CE marked standard mesh already used in routine practice, which means that adverse events are not to be expected. However, all adverse events observed during the study will be recorded and published as part of the results of this trial. Major adverse events will be reported to the principal investigator.

### Publication of study results

The results from this trial will be published in a medical journal.

### Insurance coverage of the participants

Participants will not be exposed to any additional risk beyond normal laparoscopic inguinal hernia surgery. Patients are covered by standard patient malpractice insurance.

### Cost estimate, funding and resources.

The surgeries will not incur additional costs for the operating units. Postage and copying costs are covered by the clinic´s research funds. No additional staff are needed.

No funding will be accepted from external funders (for example mesh companies)

### Trial register and confidentiality

Data collection forms are stored in folders in a locked room (Hospital research office). Data will be entered into electronic format (Excel table, SPSS table) and protected by password, data in electronic format is stored on the hospital´s computer. Data without patients´ identification numbers can be transferred to the researcher´s computer and stored when protected by password.

The trial is registered in ClinicalTrials.gov.

### References

1. HerniaSurge Group (2018) International guidelines for groin hernia management. Hernia 22(1):1-165. doi: 10.1007/s10029-017-1668-x

2. Köckerling F, Koch A, Lorenz R, Schug-Pass C, Stechemesser B, Reinpold W (2015) How Long Do We Need to Follow-Up Our Hernia Patients to Find the Real Recurrence Rate? Front Surg 16;2:24. doi: 10.3389/fsurg.2015.00024

3. Scheuermann U, Niebisch S, Lyros O, Jansen-Winkeln B, Gockel I (2017) Transabdominal Preperitoneal (TAPP) versus Lichtenstein operation for primary inguinal hernia repair – A systematic review and meta-analysis of randomized controlled trials. BMC Surg 17: 55. doi: 10.1186/s12893-017-0253-7 PMCID: PMC5424320 PMID: 28490321.

4. Li W, Sun D, Sun Y, Cen Y, Li S, Xu Q, Li Y, Qi Y, Lin Y, Yang T, Xu P (2017) The effect of transabdominal preperitoneal (TAPP) inguinal hernioplasty on chronic pain and quality of life of patients: mesh fixation versus non-fixation. Surg Endosc 31(10):4238-4243. doi: 10.1007/s00464-017-5485-1.

5. Sajid MS, Ladwa N, Kalra L, McFall M, Baig MK, Sains P (2013) A meta-analysis examining the use of tacker mesh fixation versus glue mesh fixation in laparoscopic inguinal hernia repair. Am J Surg 2013;206(1):103-11. doi: 10.1016/j.amjsurg.2012.09.003

6. Shi Z, Fan X, Zhai S, Zhong X, Huang D (2017) Fibrin glue versus staple for mesh fixation in laparoscopic transabdominal preperitoneal repair of inguinal hernia: a meta-analysis and systematic review. Surg Endosc 31(2):527-537. doi: 10.1007/s00464-016-5039-y

7. Andresen K, Fenger AQ, Burcharth J, Pommergaard HC, Rosenberg J (2017) Mesh fixation methods and chronic pain after transabdominal preperitoneal (TAPP) inguinal hernia surgery: a comparison between fibrin sealant and tacks. Surg Endosc 31(10):4077-4084. doi: 10.1007/s00464-017-5454-8.

8. Lau H. (2005) Fibrin sealant versus mechanical stapling for mesh fixation during endoscopic extraperitoneal inguinal hernioplasty: a randomized prospective trial. Ann Surg. 242 (5):670-5.

9. [M S Sajid](https://pubmed.ncbi.nlm.nih.gov/?term=Sajid+MS&cauthor_id=22449832) [^1^](https://pubmed.ncbi.nlm.nih.gov/22449832/#affiliation-1), [N Ladwa](https://pubmed.ncbi.nlm.nih.gov/?term=Ladwa+N&cauthor_id=22449832), [L Kalra](https://pubmed.ncbi.nlm.nih.gov/?term=Kalra+L&cauthor_id=22449832), [K Hutson](https://pubmed.ncbi.nlm.nih.gov/?term=Hutson+K&cauthor_id=22449832), [P Sains](https://pubmed.ncbi.nlm.nih.gov/?term=Sains+P&cauthor_id=22449832), [M K Baig](https://pubmed.ncbi.nlm.nih.gov/?term=Baig+MK&cauthor_id=22449832) (2012) A meta-analysis examining the use of tacker fixation versus no-fixation of mesh in laparoscopic inguinal hernia repair. Int J Surg.10(5):224-31.

10. Cambal M, Zonca P, Hrbaty B. (2012) [Comparison of self-gripping mesh with mesh fixation with fibrin-glue in laparoscopic hernia repair (TAPP).](https://pubmed.ncbi.nlm.nih.gov/22394041/) Bratisl Lek Listy. 113(2):103-7. doi: 10.4149/bll_2012_024.

11. Uberto Fumagalli Romario, Francesco Puccetti, Ugo Elmore, Simonetta Massaron, [Riccardo Rosati](https://pubmed.ncbi.nlm.nih.gov/?term=Rosati+R&cauthor_id=23292556) (2013) Self-gripping mesh versus staple fixation in laparoscopic inguinal hernia repair: a prospective comparison. Surg Endosc 27(5):1798-802.

12. John Ozmen [^1^](https://pubmed.ncbi.nlm.nih.gov/26523915/#affiliation-1), Vincent Choi [^1^](https://pubmed.ncbi.nlm.nih.gov/26523915/#affiliation-1), Kirsten Hepburn [^1^](https://pubmed.ncbi.nlm.nih.gov/26523915/#affiliation-1), Will Hawkins [^1^](https://pubmed.ncbi.nlm.nih.gov/26523915/#affiliation-1), Ken Loi (2015) Laparoscopic Totally Extraperitoneal Groin Hernia Repair Using a Self-Gripping Mesh: Clinical Results of 235 Primary and Recurrent Groin Hernias. J Laparoendosc Adv Surg Tech A 25(11):915-9.

13. [Dieter Birk](https://pubmed.ncbi.nlm.nih.gov/?term=Birk+D&cauthor_id=23292669) [^1^](https://pubmed.ncbi.nlm.nih.gov/23292669/#affiliation-1), [Carlos Garcia Pardo](https://pubmed.ncbi.nlm.nih.gov/?term=Pardo+CG&cauthor_id=23292669) Self-gripping Parietene and Parietex Progrip mesh laparoscopic hernia repair: have we found the ideal implant? Surg Technol Int.  2012 Dec;22:93-100.

14. Thölix AM, Kössi J, Remes V, Scheinin T, Harju J (2018) Lower Incidence of Postoperative Pain after Open Inguinal Hernia Surgery with the Usage of Synthetic Glue-Coated Mesh (Adhesix®). Am Surg. 84(12):1932-1937. PMID: 30606351

--

Translated from the original file (in Finnish) 25.01.2025.
